# The World’s Columbian Exposition

**Published:** 1893-05

**Authors:** 


### The World's Columbian Exposition.

*To the Public:*

The following official statement is of interest to the public, and can be relied upon as absolutely correct:

1. The Exposition will be opened in readiness for visitors May 1st.

2. An abundance of drinking water, the best supplied to any great city in the world, will be provided free to all. The report that a charge would be made for drinking water probably arose from the fact that hygeia water can also be had by those who may desire it at one cent a glass.

3. Ample provision for seating will be made without charge.

4. About 1,500 toilet rooms and closets will be located at convenient points in the buildings and about the grounds, and they will be absolutely free to the public. This is as large a number in proportion to the estimated attendance as has ever been provided in any exposition. In addition to these there will also be nearly an equal number of lavatories and toilet rooms of a costly and handsome character as exhibits, for the use of which a charge of five cents will be made.

5. The admission fee of 50 cents will entitle the visitor to see and enter all the Exposition buildings, inspect the exhibits, and, in short, to see everything within the Exposition grounds, except the Esquimau Village and the reproduction of the Colorado cliff dwellings. For these as well as for the special attractions on Midway Plaisance a small fee will be charged.

6. Imposition or extortion of any description will not be tolerated.

7. Free medical and emergency hospital service is provided on the grounds by the Exposition management.

8. The Bureau of Public Comfort will provide commodious free waiting-rooms, including spacious ladies' parlor and toilet rooms in various parts of the grounds.

H. N. Higginbotham, President.

---

Chicago, April 17, 1893.

*Editor of Dental Register, Cincinnati, Ohio.*

Dear Doctor: Owing to a change in the time of meeting of the World's Columbian Dental Congress, it seemed a necessity to make a change in the time of meeting of the American Dental Association, and at the request of the officers of both the American Dental Association and the World's Columbian Dental Congress, I communicated with the officers of the former and the vote was unanimous for changing the time of meeting of the American Dental Association.

Accordingly we give notice that the meeting of the American Dental Association will be held in Chicago, August 12, instead of August 15.

By order of the Executive Committee.

J. N. Crouse, Chairman.

